# Preparation of Cuprous Oxide Mesoporous Spheres with Different Pore Sizes for Non-Enzymatic Glucose Detection

**DOI:** 10.3390/nano8020073

**Published:** 2018-01-29

**Authors:** Jingwen Ma, Jun Wang, Min Wang, Guoliang Zhang, Wenchao Peng, Yang Li, Xiaobin Fan, Fengbao Zhang

**Affiliations:** Lab of Advanced Nano-Structures & Transfer Processes, Department of Chemical Engineering, Tianjin University, Tianjin 300354, China; majingwen@tju.edu.cn (J.M.); junwang@tju.edu.cn (J.W.); wangmin0016@126.com (M.W.); zhangguoliang@tju.edu.cn (G.Z.); wenchao.peng@tju.edu.cn (W.P.)

**Keywords:** cuprous oxide, mesoporous spheres, non-enzymatic sensor, glucose, internal diffusion

## Abstract

Mass transfer plays a significant role in a sensor’s performance, because the substrate can be detected only when it contacts with the active catalytic surface. In this work, cuprous oxide mesoporous nanospheres (Cu_2_O MPNS) with different pore size distributions are fabricated and applied as electrocatalysts for glucose detection. The small pore Cu_2_O (SP-Cu_2_O, mean pore size of 5.3 nm) and large pore Cu_2_O (LP-Cu_2_O, mean pore size of 16.4 nm) spheres are prepared by the template method and an etching treatment. The obtained two kinds of Cu_2_O MPNS exhibit high porosity with a similar specific surface area of 61.2 and 63.4 (m^2^·g^−1^), respectively. The prepared Cu_2_O MPNS are used to construct an electrochemical non-enzymatic glucose sensor. The results show that the LP-Cu_2_O exhibits better performance than SP-Cu_2_O, which illustrates that the internal diffusion takes a great impact on the performance of the sensor. The LP-Cu_2_O modified electrode possesses a high and reproducible sensitivity of 2116.9 μA mM^−1^·cm^−2^ at the applied potential of 0.6 V with a wide detection range of 0.003–7.8 mM and a low detection limit of 0.42 μM.

## 1. Introduction

Glucose biosensors, which play an important role in the monitoring of blood glucose, have become a reliable tool for the treatment and control of diabetes [1,2,3]. The majority of amperometric biosensors for glucose detection focus on two major types: enzymatic and non-enzymatic sensors [4]. Due to the high sensitivity and good selectivity, most glucose biosensors focus on glucose oxidase. However, they suffer from poor reproducibility, a complicated immobilization process, and thermal and chemical instability because of the intrinsic properties of the enzymes. To solve this problem, plentiful efforts have been made to the research of non-enzymatic glucose biosensors. As a member of glucose sensors, non-enzymatic glucose sensors based on the direct electrocatalysis of glucose have recently drawn great attention due to their efficient glucose detection sensitivity and stability [2,5,6,7]. Owing to the high electrocatalytic ability, materials based on noble metals, such as Pt [8], Pd [9], and Ag [10], transition metals, such as Cu [11], Ni [12,13], Mn [14], and Co [15], and their oxides [16], or polymers, such as polyaniline [17], have been found to be active electrocatalytic materials for glucose biosensors. Among them, cuprous oxide (Cu_2_O), an important semiconductor with a band gap of 2.2 eV [18], has exhibited promising applications in the field of sensing, which is attributed to its minimal cost, stability, and significant catalytic activity [19,20,21,22]. Recently, in order to introduce larger surface areas and more catalytically active sites for the electrocatalytic reactions, porous Cu_2_O nanomaterials have been prepared and used to build biosensors with improved performances [23,24,25,26].

However, the efficiency of electrochemical sensors is significantly influenced by the substrate adsorption and charge transfer on the electrode surface, because the glucose molecules can be oxidized only when they contact with the active sites. As a consequence, the performances of these electrocatalytic sensors may be subject to mass transfer limitations, especially for the porous systems. In principle, the mass transfer resistance comes from both external and internal mass transfer [27]. Similar to the reactions with normal porous catalysts, the mass transfer of the substrate first occurs from the bulk fluid to the external surface of the catalyst, then diffuses from the external surface into and through the pores within the pellet [28,29]. The external diffusion can be neglected with vigorous stirring, hence the internal diffusion will play a significant role in the detection ability of the biosensor [30]. Therefore, promoting the internal diffusion rate may facilitate the sensitivity of the biosensors. Altering the pore size of the materials can adjust the internal diffusion rate due to the fact that the internal diffusion rate has a proportional relationship with the pore size. However, there is little research about the influence of different pore sizes on glucose sensors.

**Scheme 1:**
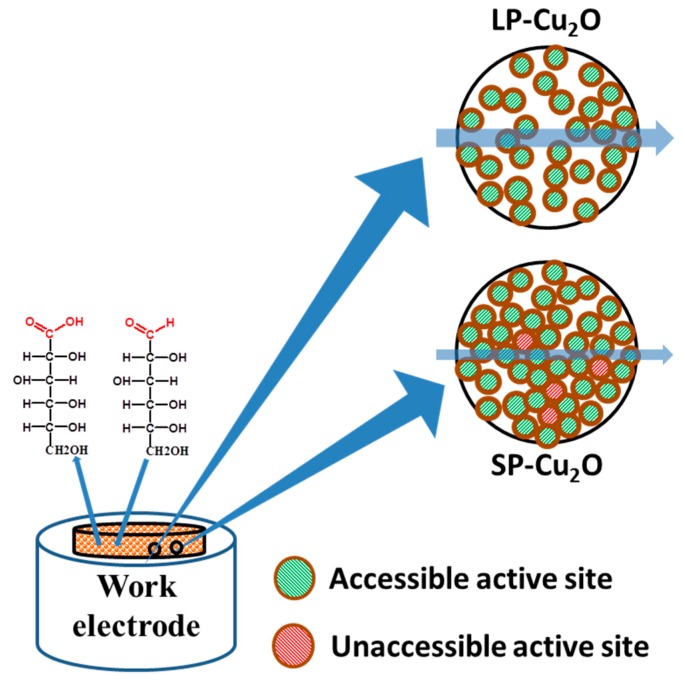
Illustration of glucose oxidation using mesoporous Cu_2_O spheres with different internal diffusion rates as electrocatalysts. LP-Cu_2_O: large pore Cu_2_O; SP-Cu_2_O: small pore Cu_2_O.

Herein, we report the fabrication of mesoporous Cu_2_O nanospheres with different pore sizes and their performances on the glucose sensors (Scheme 1). Comparative studies reveal that a Cu_2_O sphere with a relatively larger pore size shows higher activity, a wider detection range, and a lower detection limitation for glucose detection, demonstrating the important role of internal diffusion on their performances for glucose detection.

## 2. Materials and Methods

The porous Cu_2_O nanospheres with a small pore size (SP-Cu_2_O) were prepared according to the template synthetic process with some modification. Firstly, 0.5 g triblock copolymer pluronic P123 (MW 5800) was dissolved in 40 mL deionized water and stirred for at least 5 h. Then, 3 mL of 0.2 M Cu(NH_3_)_4_^2+^ solution (10:1, NH_3_:Cu^2+^) was transferred into the P123 solution under constant stirring. After 40 min, 6 mL of 0.6 M ascorbic acid solution was added dropwise into the above mixture. After stirring for 40 min, the resulting precipitate was collected by centrifugation, washed with ethanol at 333 K overnight to remove the P123, and finally dried in vacuum at 333 K for 4 h.

The porous Cu_2_O nanospheres with a large pore size (LP-Cu_2_O) were prepared using the etching method. Typically, 5 mg of the as-prepared SP-Cu_2_O was added into 10 mL of the ethanol solution containing 1.2 mg·mL^−1^ of L-proline under constant stirring. After 8 h, the precipitate was collected by centrifugation, washed several times with distilled water and ethanol, and finally dried in vacuum at 333 K for 4 h.

An X-ray power diffraction (XRD) analysis was carried out using a Bruker advance-D8 power diffractometer (Karlsruhe, Germany) with Cu Kα radiation (*λ* = 0.154178 nm). Field-emission scanning electron microscopy (FESEM) images were obtained on a Hitachi S-4800 microscope (Tokyo, Japan) operating at 5 kV. X−ray photoelectron spectrum (XPS) was conducted on a PHI 1600 spectrometer (PerkinElmer, Shelton, CT, USA) to analyze the surface composition. For transmission electron microscopy (TEM) and high-resolution transmission electron microscopy (HRTEM), the structures were imaged with a JEM-2011F microscope (JEOL, Tokyo, Japan) with an accelerating voltage of 200 kV and a JEOL JEM-3010 microscope (JEOL, Tokyo, Japan) operated at 200 kV, respectively. The nitrogen adsorption-desorption isotherms were obtained with ASAP2010 apparatus (Micrometritics, Atlanta, GA, USA) at 77 K. The surface areas of the obtained samples were calculated by the Brunauer–Emmett–Teller (BET) method, and the pore size distribution curve was derived from the adsorption using the Barrett–Joyner–Halenda (BJH) theory.

Electrochemical measurements were conducted using an electrochemical workstation (CHI 660E, Chenhua, Shanghai, China) connected to the computer for glucose detection. All of the electrochemical measurements were carried out using a conventional three-electrode system with a magnetic stirrer. Platinum wire was used as the counter electrode, saturated calomel electrode was used as the reference electrode, and a Cu_2_O mesoporous nanospheres (MPNS)-modified glassy carbon electrode (GCE) was used as the working electrode. The GCE was polished with alumina slurries and cleaned by sonication in the deionized water and ethanol, respectively. Four milligrams of the prepared Cu_2_O sample was added in 2 mL of 5 wt % Nafion solution and dispersed in an ultrasonic bath for 30 min. Then, a 10 μL dispersion (2 mg·mL^−1^) was dropped onto the GCE’s surface and dried in air overnight to afford a Cu_2_O-modified GCE.

## 3. Results and Discussion

**Figure 1:**
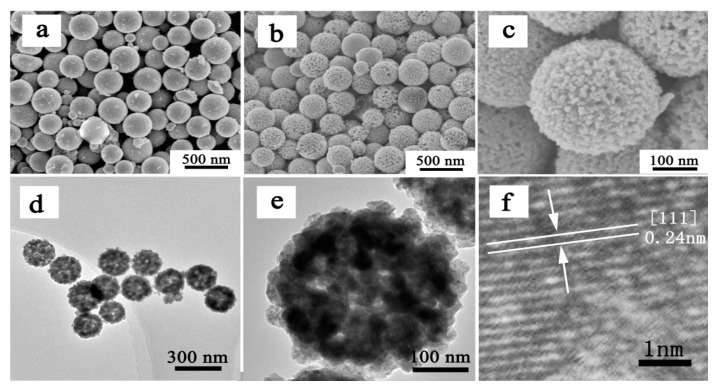
SEM (**a**) image of SP-Cu_2_O; SEM (**b**,**c**) and TEM (**d**,**e**) images of LP-Cu_2_O; HRTEM (**f**) image of the LP-Cu_2_O.

The morphologies of the samples were characterized by field emission scanning electron microscopy (FESEM) and transmission electron microscopy (TEM). Figure 1a shows the FESEM image of SP-Cu_2_O. Figure 1b shows FESEM images of LP-Cu_2_O. It can be found that both the SP-Cu_2_O and LP-Cu_2_O spheres are uniformly dispersed and with an average diameter of 320 nm. Moreover, the distinct widespread pores could be observed on the SEM image of LP-Cu_2_O, which demonstrates its large porous property. The magnified SEM image of a typical sphere (Figure 1c) illustrates its mesoporous structure feature. As shown in Figure S1, the mesoporous structure is destroyed by extending the etching time to 10 h. Figure 1d,e and Figure S2 are the corresponding TEM images of the LP-Cu_2_O and SP-Cu_2_O, which illustrate that the Cu_2_O spheres have a porous structure. The observed lattice fringes in the HRTEM image (Figure 1f) show a clear lattice spacing of 0.24 nm, corresponding to the distance of the (111) planes of Cu_2_O.

**Figure 2:**
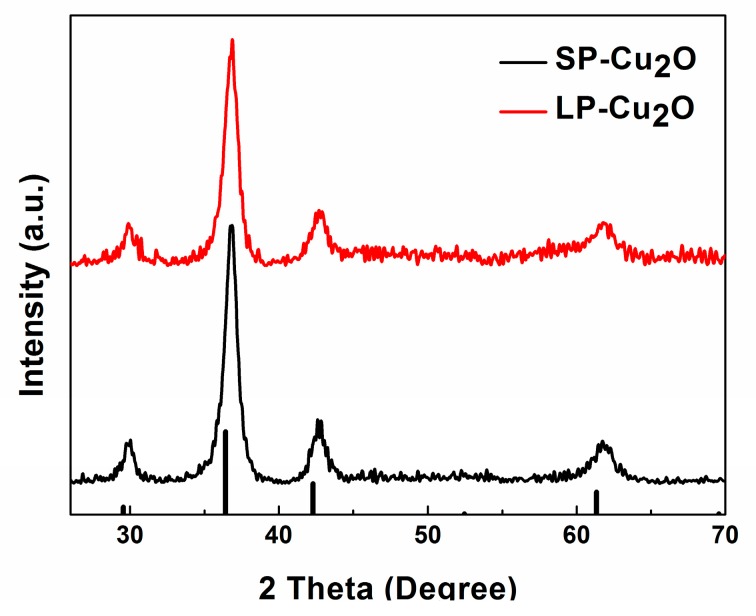
XRD patterns of the as-prepared SP-Cu_2_O and LP-Cu_2_O.

Figure 2 shows the X-ray diffraction (XRD) patterns of SP-Cu_2_O and LP-Cu_2_O, in which can be seen four sharp peaks at 2*θ* = 30, 37, 42.6, and 62.4°, corresponding to the (110), (111), (200), and (220) planes of Cu_2_O (JCDPS No. 05-0667) [31]. There is no obvious peak showing the formation of any phase of copper (Cu) or copper oxide (CuO), which indicates the high purity of the prepared samples. Additionally, XPS is employed to determine the surface state. Figure S3 displays the XPS spectrum of Cu 2p. The peaks at 952.1 and 932.3 eV are assigned to Cu 2p_1/2_ and Cu 2p_3/2_ of Cu_2_O, respectively. No shake-up satellite peaks are found at 940–945 eV, indicating that Cu (I) is dominant in the LP-Cu_2_O.

**Figure 3:**
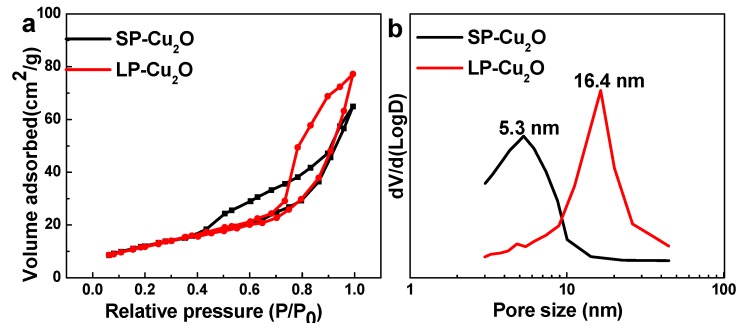
Nitrogen adsorption-desorption isotherms (**a**) and the corresponding Barrett–Joyner–Halenda (BJH) pore size distribution curves (**b**) of the SP-Cu_2_O and LP-Cu_2_O.

The N_2_ adsorption-desorption isotherms are measured to testify to the porosity of the as-prepared Cu_2_O spheres. Figure 3a shows the N_2_ adsorption-desorption isotherms of SP-Cu_2_O and LP-Cu_2_O, which indicate that the two isotherms belong to type IV hysteresis loops, providing the evidence for the mesoporous structure. The BET specific surface area of the SP-Cu_2_O is 61.2 m^2^·g^−1^, which is almost identical with that of the LP-Cu_2_O (63.4 m^2^·g^−1^), indicating that the surface area is slightly increased after the etching treatment. This may be due to the crystal size of LP-Cu_2_O, which as calculated from XRD, is slightly decreased after etching [32]. Figure 3b displays the pore size distribution curves. The mean pore size of LP-Cu_2_O is 16.4 nm, which is much larger than that of SP-Cu_2_O (5.3 nm). Nevertheless, a weak peak appears at about 5 nm from the BJH curve of the LP-Cu_2_O in Figure 3b, which may be ascribed to a small part of unetched pores.

Generally, the diffusion of liquid in the pore of the porous catalyst is in accordance with Fick-diffusion according to mass transfer theory [33]. The effective diffusion coefficient (*D*_ABP_) can be defined as follows:

(1) $$D_{\mathrm{ABP}}=\frac{\varepsilon \;D_{\mathrm{AB}}}{\tau }$$

The pore size and the void fraction (ε) have a proportional relationship, while it has an inverse relationship with the twist factor (*τ*). *D*_AB_ is constant in a certain solution system. On the basis of Equation (1), the *D*_ABP_ value of LP-Cu_2_O is larger than that of SP-Cu_2_O. Therefore, the liquid diffuses faster in the pores of LP-Cu_2_O than in those of SP-Cu_2_O.

**Figure 4:**
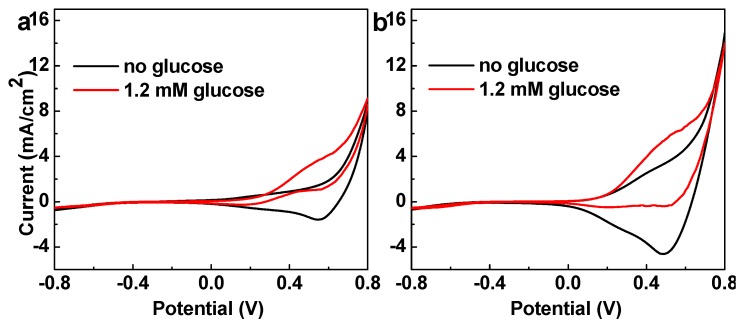
Cycle voltammetry curves (CVs) of the SP-Cu_2_O-modified electrode (**a**) and LP-Cu_2_O-modified electrode (**b**) in the absence and presence of 1.2 mM glucose in 0.1 M KOH solution at a scan rate of 50 mV s^−1^.

The non-enzymatic glucose sensor is created by direct deposition of SP-Cu_2_O or LP-Cu_2_O on the surface of a glassy carbon electrode (GCE). Figure 4 depicts the cycle voltammetry curves (CVs) of the SP-Cu_2_O and LP-Cu_2_O in 0.1 M KOH solution with and without the addition of glucose at a scan rate of 50 mV s^‒1^. Comparing the CVs in Figure 4a, a relatively larger current signal can be observed in Figure 4b, which illustrates that the LP-Cu_2_O exhibits a higher activity towards glucose electro-oxidation. The enhanced electrocatalytic activity may be attributed to the kinetic effect by the improved internal diffusion rate of the LP-Cu_2_O, because with the improved internal diffusion ability there will be more catalytically active sites, which could be attained by the glucose molecules.

**Figure 5:**
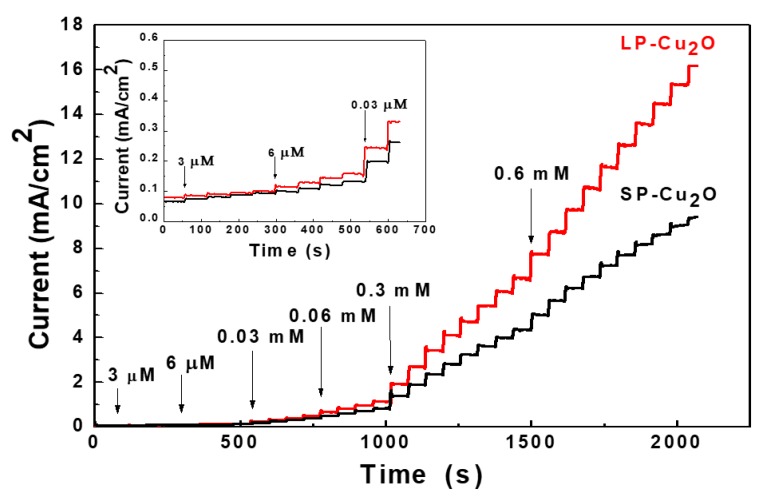
Amperometric response of SP-Cu_2_O and LP-Cu_2_O electrodes to successive additions of glucose at 0.6 V. Inset: a magnification view of the current response toward a low concentration of glucose solution.

The amperometric response of the Cu_2_O MPNS modified electrodes towards glucose oxidation is conducted at 0.6 V for the successive addition of various concentrations of glucose solution with violent stirring. Upon the injection of glucose solution (Figure 5), the modified electrodes exhibit an obvious increase of current density to the change of glucose concentration. Both catalysts can acquire 97% of steady-state current within 5 s, indicating the sensitive and rapid response behavior of SP-Cu_2_O and LP-Cu_2_O towards a glucose electro-oxidation reaction. Moreover, the current response of the LP-Cu_2_O for an equal concentration of glucose solution is obviously larger than that of the SP-Cu_2_O.

**Figure 6:**
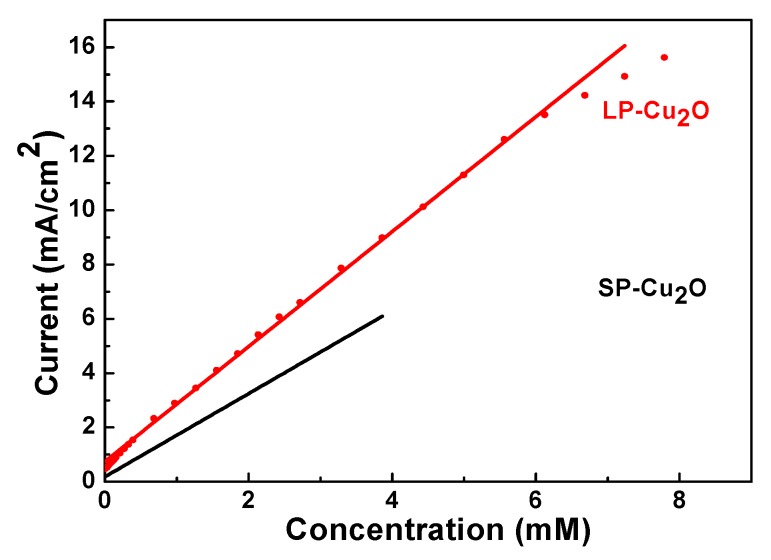
The corresponding calibration curves of SP-Cu_2_O and LP-Cu_2_O for glucose detection.

Figure 6 illustrates the linear relationship between the current response and the glucose concentration of SP-Cu_2_O- and LP-Cu_2_O-modified electrodes, which shows that both catalysts exhibit a good linear relationship towards the concentration of the glucose. The linear regression equations acquired from the amperometric response of SP-Cu_2_O and LP-Cu_2_O are *J(y*) = 0.6775 + 1.5314*C*(*x*) and *J*(*y*) = 0.7405 + 2.1169*C*(*x*), respectively. Hence, the factor *J* refers to the current response density and *C* is the concentration of the glucose. The sensitivity of the LP-Cu_2_O (2116.9 μA mM^−1^ cm^−2^), which is calculated from the calibration curves, is about 1.38-fold greater than that of the SP-Cu_2_O (1531.4 μA mM^−1^ cm^−2^). The detection limit of LP-Cu_2_O (0.42 μM) is lower than that of the SP-Cu_2_O (0.63 μM), and the linear range of LP-Cu_2_O (up to 7.234 mM) is much wider than that of the SP-Cu_2_O (up to 3.861 mM). The above results indicate that in comparison with SP-Cu_2_O, the LP-Cu_2_O exhibits higher electrocatalytic activity towards glucose electro-oxidation with higher sensitivity and a lower detection limit. The catalytic activity is proportional to the effective catalytic surface area where the glucose can contact. We use the same amount of SP-Cu_2_O and LP-Cu_2_O to modify the electrodes. The total surface area is considered equal on the basis of the almost same BET specific surface area of the two samples. Therefore, supposing that all of the surface is used as the active surface, the sensor effect should be same. The effect of the external diffusion can be neglected, which is owing to the fact that the experiments are conducted with under a sufficient stirring condition. Therefore, the internal diffusion plays a vital role on the sensors’ performance. Compared with SP-Cu_2_O, LP-Cu_2_O has the same surface area but an improved sensor performance due to the higher internal diffusion rate of the LP-Cu_2_O. The results prove that the fabrication of a large-pore electrocatalyst can improve the efficiency of mass transfer so as to improve the electrocatalytic activity. Additionally, the comparison of the LP-Cu_2_O and other reported Cu-based glucose sensors is summarized in Table S1. Obviously, the sensor in this work exhibits good analytical properties, which are comparable to or even higher than those of other reported Cu-based electrochemical sensors for glucose.

**Figure 7:**
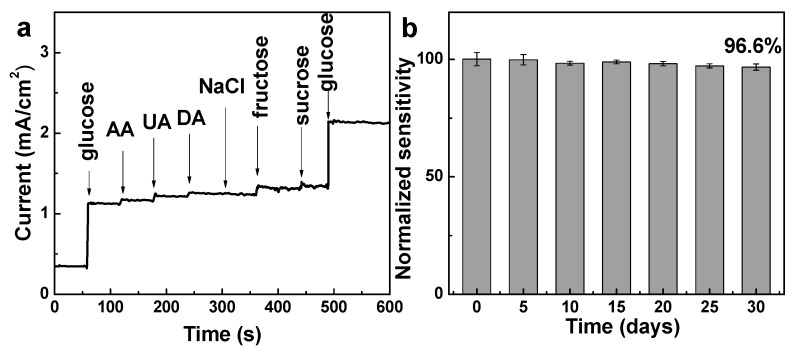
(**a**) Amperometry response of LP-Cu_2_O upon successive addition of 0.3 mM glucose, 0.1 mM ascorbic acid (AA), 0.1 mM uric acid (UA), 0.1 mM dopamine (DA), 0.1 mM NaCl, 0.1 mM fructose, 0.1 mM sucrose, and 0.3 mM glucose at 0.6 V; (**b**) Normalized sensitivity of the LP-Cu_2_O-based electrode sensor for glucose detected every 5 days over 1 month.

Another important analytical factor for an electrochemical sensor is its ability to discriminate the interfering species having similar electroactivities on the electrocatalyst. So, the selectivity of the as-prepared biosensor is evaluated against some interfering compounds, including dopamine (DA), ascorbic acid (AA), uric acid (UA), fructose, and sucrose, which usually co-exist with glucose in human blood serum [4,34]. The blood glucose level of a normal human body is between 4 and 7 mM, which is approximately 30 to 50 times higher than that of the interferences. The interfering species with only 5–10% of glucose concentration are applied in the selectivity tests in the previous studies. In this work, a higher concentration of DA, AA, UA, fructose, and sucrose are used to test the selectivity of the catalyst. Figure 7a shows the amperometric response of the LP-Cu_2_O-modified electrode toward the successive addition of 0.3 mM glucose, 0.1 mM ascorbic acid (AA), 0.1 mM uric acid (UA), 0.1 mM dopamine (DA), 0.1 mM NaCl, 0.1 mM fructose, 0.1 mM sucrose, and 0.3 mM glucose in a stirred 0.1 M KOH solution at the applied potential of 0.6 V. It can be seen that the current responses to interfering species are rather weak compared to that from glucose oxidation, suggesting the excellent selectivity of the LP-Cu_2_O-modified electrode. Therefore, the LP-Cu_2_O-modified electrode is a promising candidate in practical application for glucose detection.

The long-term storage stability of the electrode is characterized by storing under ambient conditions and studying the response intermittently. As shown in Figure 7b, the activity of the LP-Cu_2_O-modified electrode is nearly unchanged within 10 days, and retains 97.2% of its original sensitivity after 30 days. The reproducibility of the sensor is also evaluated by preparing three electrodes through the same method. A relative standard deviation (RSD) of 3.88% is acquired, which suggests the good reproducibility of the LP-Cu_2_O-modified electrode.

## 4. Conclusions

In summary, different pore-size Cu_2_O nanospheres with a high specific surface area and a uniformly porous spherical morphology are successfully synthesized by the template method with an etching treatment. In the electrochemical measurements for glucose detection, the LP-Cu_2_O modified electrode exhibits better electrocatalytic activity compared with the SP-Cu_2_O modified electrode. The results show that internal diffusion rate plays a vital role in the performance of the sensor. The obtained LP-Cu_2_O biosensor exhibits a low detection limit, good sensitivity, excellent selectivity, good stability, reproducibility, and a wide detection range (0.003 to 7.8 mM) due to the improved internal diffusion and electron transfer ability.

## Supplementary Materials

The following are available online at http://www.mdpi.com/2079-4991/8/2/73/s1. Figure S1: SEM image of LP-Cu_2_O with longer etching time; Figure S2: TEM image of SP-Cu_2_O; Figure S3: XPS spectrum of Cu 2p for LP-Cu_2_O; Table S1: Comparison of similar non-enzymatic glucose sensors.
