# Supplementary material for: Preparation of Cuprous Oxide Mesoporous Spheres with Different Pore Sizes for Non-Enzymatic Glucose Detection
Source: Nanomaterials (Basel). 2018 Jan 29;8(2):73. doi: 10.3390/nano8020073 (PMC5853705; doi:10.3390/nano8020073)
Supplement: Supplementary file 1 [file nanomaterials-08-00073-s001.pdf]

Supporting Information

Preparation of cuprous oxide mesoporous spheres with different pore size for non-enzymatic glucose detection

Jingwen Ma, Jun Wang, Min Wang, Guoliang Zhang, Wenchao Peng, Yang Li*, Xiaobin Fan*, Fengbao Zhang*

Lab of Advanced Nano-structures &Transfer Processes, Department of Chemical Engineering, Tianjin University, Tianjin 300354, China

* Correspondence: liyang1895@tju.edu.cn; xiaobinfan@tju.edu.cn; fbzhang@tju.edu.cn; Tel: +86-22-27890090

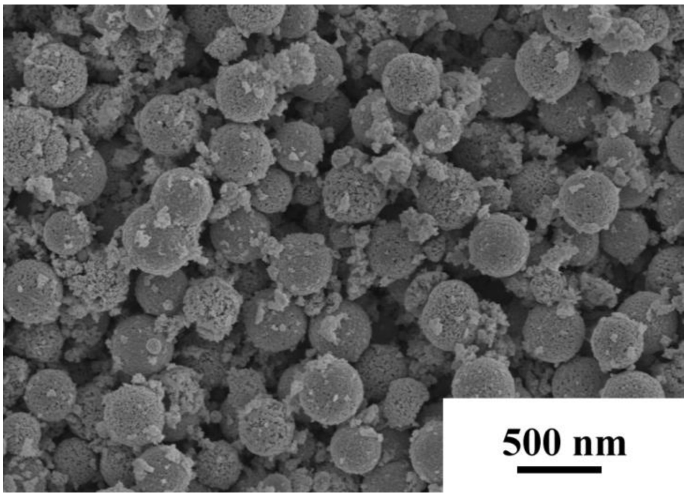

Figure S1. SEM image of LP-Cu_2O with longer etching time.

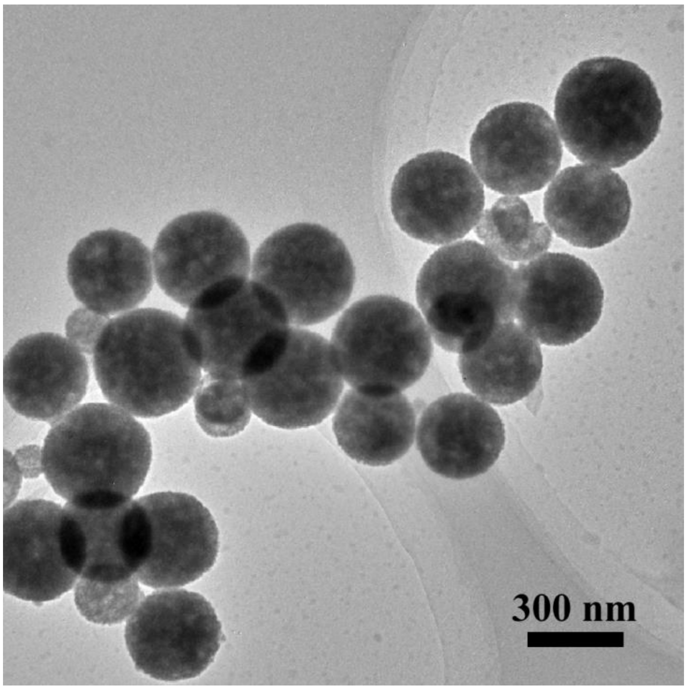

Figure S2. TEM image of SP-Cu_2O.

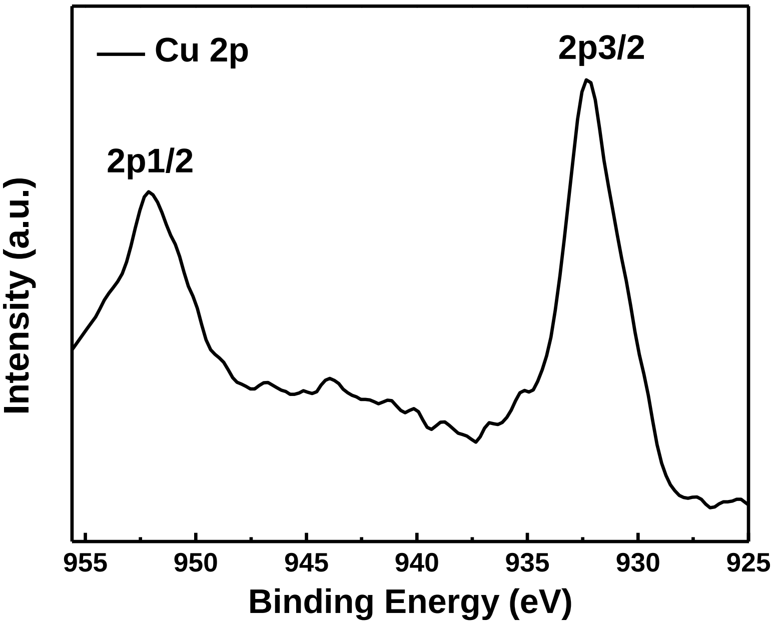

Figure S3. XPS spectrum of Cu 2p for LP-Cu_2O.

Table S1. Comparison of similar non-enzymatic glucose sensors.

**Materials**	**Linear range (mM)**	**Sensitivity (μA mM^{-1} cm^{-2})**	**Detection Limit (μM)**	**Reference**
**Au@Cu_2O**	0.05-2	715	18	1
**Octahedral Cu_2O**	0.1-5	293.893	5.11	2
**Cu_2O/graphene**	0.01-3	1330.05	0.36	3

**Hollow Cu_2O**	0.00125-0.0375	2038.2	0.41	4
**RGOs-Cu_2O**	0.01-6	2619	0.05	5
**LP-Cu_2O**	0.003-7.8	2116.9	0.42	This work

1. Su, Y.; Guo, H.; Wang, Z.; Long, Y.; Li, W.; Tu, Y. Au@Cu 2 O core-shell structure for high sensitive non-enzymatic glucose sensor. *Sens Actuators, B* **2018**, *255*, 2510-2519.
2. Tang, L.; Lv, J.; Kong, C.; Yang, Z.; Li, J. Facet-dependent nonenzymatic glucose sensing properties of Cu2O cubes and octahedra. *New J Chem* **2016**, *40*, 6573-6576.
3. Yazid, S. N. A. M.; Isa, I. M.; Hashim, N. Novel alkaline-reduced cuprous oxide/graphene nanocomposites for non-enzymatic amperometric glucose sensor application. *Materials Science and Engineering: C* **2016**, *68*, 465-473.
4. Cao, H.; Yang, A.; Li, H.; Wang, L.; Li, S.; Kong, J.; Bao, X.; Yang, R. A non-enzymatic glucose sensing based on hollow cuprous oxide nanospheres in a Nafion matrix. *Sens Actuators, B* **2015**, *214*, 169-173.
5. Zhou, D.-L.; Feng, J.-J.; Cai, L.-Y.; Fang, Q.-X.; Chen, J.-R.; Wang, A.-J. Facile synthesis of monodisperse porous Cu2O nanospheres on reduced graphene oxide for non-enzymatic amperometric glucose sensing. *Electrochim Acta* **2014**, *115*, 103-108.
